# The Future of Surgical Research

**DOI:** 10.1371/journal.pmed.0010013

**Published:** 2004-10-19

**Authors:** Robert J Weil

## Abstract

Surgeons seem to love publishing case series, which are of limited usefulness. How can we encourage them to do randomized clinical trials?

In 1996, Richard Horton, editor of the *Lancet,* chastised much of current surgical research and, in particular, questioned the usefulness of the case series as a predominant form of communication among surgeons [1]. He asked a poignant question: “Does surgical research have a future?” Nearly a decade later, it is important for surgeons and non-surgeons alike to revisit Horton's challenge.

## Why Surgeons Favor Case Series

Randomized controlled trials (RCTs) have become the pillar of clinical research. Such trials attempt to obtain an unbiased randomization of patients with respect to known and unknown baseline conditions and to assess the effects of an intervention. However, only a minority of surgical studies involve a valid randomization scheme. The case series remains a favored method of clinical investigation in surgery.

Case series are easy to perform, require less resources in terms of personnel and funds, can be performed at a single center, and, for many surgeons, represent a means to illustrate their surgical method and skills. In many instances, case series also serve as valuable intellectual background for future clinical or scientific work. For example, consider Dennis Burkitt's report on jaw tumors in African children, Alfred Blalock's initial efforts in cardiac surgery, or, more recently, Starzl and colleagues' observations, in a small collection of patients, of donor leukocyte chimerism, whereby recipients acquire tolerance to foreign donor cells. In all three cases, the authors' work led to powerful shifts in our understanding of the biology and treatment of disease [2,3,4]. All were case reports or case series—but under the current paradigm adopted by most journals and evidence-based databases, they would not be valued [5,6,7].[Fig pmed-0010013-g001]

**Figure pmed-0010013-g001:**
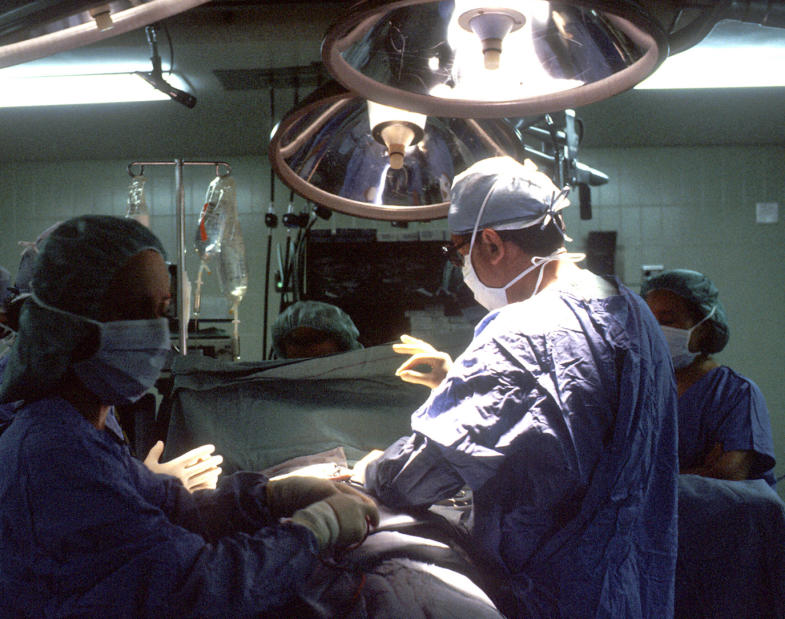
Surgical research needs to move from case series to RCTs (Photo by Linda Bartlett, National Cancer Institute)

## Barriers to Surgical RCTs

There are many reasons why RCTs in surgical patients may be more difficult to perform than those in non-surgical patients. One of the most important—though least understood—is that the complexities of human disease in surgical patients makes them a more difficult group to study. Surgical patients are often heterogeneous in many more ways than non-surgical patients. So it would be inherently easier, for example, to study a new medication for generally healthy young adults with essential hypertension than a surgical technique for older patients with hepatic failure needing transplantation.

In addition, while there may be value in studying patients from multiple centers, there may be important differences in the skill levels of different surgeons, either between centers or across the country. For example, the skill levels of surgeons in trials of carotid endarterectomy may be greater than those across the surgical community as a whole. This makes the applicability of some surgical RCTs to the wider community less certain than trials of medical therapies.

So when it comes to surgical research, for both researchers and funding agencies, it is easier to grapple with a difficult, but ultimately soluble, basic science question than to face the uncertainty of clinical research. Investigators understand these implicit issues and trim their sails accordingly.

## Improving the Rigor of Research

Nonetheless, too much surgical work is conducted in the less rigorous format of the case series. What can and should be done to improve the rigor of surgical investigation? It would seem that improvements are required from within and beyond the surgical world.

First, as Horner observed, and several eminent surgeons have since agreed, reforms must begin within the field itself [1,5,6,7]. Both during surgical training and in the early years of faculty development, surgeons must obtain a thorough grounding in the principles of basic research and proper clinical investigation.

Second, surgeons must establish firm and friendly relations with biostatisticians so that the latter may play a strong role in helping to develop adequately powered studies that can answer critical questions raised by new therapies and techniques. This is an especially acute need in an accelerating age of targeted therapies and disease biomarkers.

Third, surgeons must re-engage in the clinical research enterprise and resume leadership roles in local and national clinical trials that involve surgical patients. In the United States, for example, an important step in this regard has been the establishment of the American College of Surgeons Oncology Group, which invites surgeons from all sectors, including private practice, to become active participants in well-designed, multi-institutional trials [5]. Similar efforts are needed on a global level.

Finally, similar to the pressures faced by their colleagues elsewhere in academia, surgeon clinician-investigators must be nurtured, protected, and valued by their colleagues and medical administrators. The financial health of academic medical centers relies heavily on the generation of clinical revenue, which in many centers falls disproportionately on the shoulders of surgeons. New paradigms for revenue generation and funding of clinical research are needed.

## Funding for Surgical Research

Beyond the walls of the academic medical center, there also needs to be greater recognition of the value of scientifically sound surgical research and clinical investigation. However, the National Institutes of Health (NIH), the major source of biomedical funding in the United States, continues to convey a less welcoming attitude toward surgical research than toward other types of clinical or basic science[8,9].

At the NIH, the principal instrument for performing peer review and making grant funding decisions is the study section, composed of about 10–20 members with expertise in a given field. There are few study sections devoted to surgically oriented clinical research and only two study sections (from among more than 100) in which surgeons make up even a reasonable minority of the committee members [8]. In comparison to those in other clinical departments, surgical grant proposals are less likely to be funded, and awards, when funded, are smaller [8].

Funding agencies need to recognize the importance of the surgical endeavor to modern medicine.

Surgical research is also impeded by processes affecting other types of research as well. The number of researchers under 35 years of age receiving a first RO1 grant, the main NIH mechanism for external funding, in any field, is below 4%. The average age of initial funding for US physicians is about 44 years, and shows a trend toward advancing age that has progressed significantly in the past two decades. Thus, the NIH appears to reward experience and proven results very heavily, which may stifle innovation and likely serves as an innate barrier for younger physician-investigators contemplating research careers [9].

To help correct for this worrisome trend, the NIH created the “K” award system—career development grants designed to help starting researchers gain the experience needed to compete for RO1 grants. However, nearly 40% of the clinicians who receive KO8 awards never apply for RO1 funding [10], which suggests that the overall support—both explicit and implicit—for clinical research at the institutional and funding levels is inadequate.

Finally, outside the US, surgeons face similar, if not greater problems. This bodes poorly for countries where the cost of evaluating new therapies and technologies may be an unaffordable luxury. These challenges to the surgical research enterprise are therefore global issues and should merit the attention of surgeons, medical institutions, and funding agencies in all countries.

## The Future

What can be done? On the national and international level, funding agencies need to recognize the importance of the surgical endeavor to modern medicine. Recently, in the US the NIH unveiled a “roadmap” (http://nihroadmap.nih.gov) designed to provide “new pathways to discovery.” Clear, careful, scientific surgical investigation must be part of this roadmap, although it is not specifically mentioned. Outreach efforts to include surgeons in a variety of study sections should be made to ensure that important insights into the pathophysiology and treatment of disease, with which surgeons are concerned on a daily basis, are not overlooked. Additional efforts are needed to improve funding for clinical research, both for individuals at early stages of their careers and for multi-disciplinary clinical research and clinical trials. Locally, and individually, surgeons must join efforts to improve the clinical research enterprise by including training in clinical investigation at an early stage in medical school and during surgical residency training, fostering the careers of young surgeon-investigators through committed, protected time, participating in local and national clinical research groups, and recognizing that development as a clinical researcher takes time—many years in fact.

These efforts may help ensure that surgical research is a vital part of the future of medicine and that it leads to the kind of high-quality work that shapes and remodels the face of medicine. To foster these efforts, surgeons must change and adapt to the currents of modern medical research. If this is successful, the case series will become the occasional rather than the common form of surgical communication. And surgeons, other clinicians, and, most importantly, basic scientists will be better able to take advantage of the new avenues of biomedical science opening before us.

But the case series will always represent one important tool for early studies or uncommon conditions. It remains true that while the method one uses influences the answer one receives, it can be just as important to ask the right questions, which can be asked even in a series of one patient [11]. And surely that is the place one must begin.

## Abbreviations

NIH: National Institutes of Health
RCT: randomized controlled trial
